# The Pregnancy Environment and Lifestyle Study (PETALS): a population-based longitudinal multi-racial birth cohort

**DOI:** 10.1186/s12884-017-1301-0

**Published:** 2017-04-17

**Authors:** Yeyi Zhu, Monique M. Hedderson, Juanran Feng, Ashley A. Mevi, Assiamira Ferrara

**Affiliations:** Kaiser Permanente Division of Research, 2000 Broadway, Oakland, CA 94612 USA

**Keywords:** Birth outcomes, Cohort, Environment, Gestational diabetes, Phenols, Pregnancy

## Abstract

**Background:**

Increasing recognition has been received regarding the proven and suggested links between multi-level environmental exposures on a broad scale (e.g., chemical, clinical, behavioral, physical and social) and health deficits originated from the critical window of development. However, such prospective human data are limited. In 2016, the National Institutes of Health funded 35 centers comprising 84 extant cohorts for the Environmental Influences on Child Health Outcomes (ECHO) pediatric cohorts program. The Pregnancy Environment and Lifestyle Study (PETALS) is one of the cohorts at the participating centers of Kaiser Permanente Northern California (KPNC).

**Methods:**

PETALS was originally funded by the National Institute of Environmental Health Sciences to establish a longitudinal birth cohort of 3,350 mother-infant pairs and conduct a nested case–control study of 300 women with gestational diabetes (GDM) and 600 matched controls to investigate the associations between phenol exposures in first and second trimesters and GDM risk and the related outcome of infant macrosomia. This paper describes the prospective cohort design of PETALS, current research activities, and cohort profile of enrolled women who delivered as of February 2016. Women are enrolled from the KPNC membership. Fasting blood draw, urine collection, anthropometric measurements, and questionnaires on health history and lifestyle are completed at baseline and follow-up clinic visits with targeted windows of 10–13 and 16–19 weeks of gestation, respectively. Further, women’s clinical and health assessments before and after the index pregnancy in addition to their children’s birth outcomes and health information can be abstracted from electronic health records, allowing future follow-up. Study data could also be linked and extended to a myriad of additional observational data including environmental and area-level databases and census data.

**Discussion:**

In this racially- and ethnically-diverse pregnancy cohort, the generated biospecimen and data repository will establish a comprehensive framework which may provide unique opportunities to address a multitude of research questions on the intrauterine environment and adverse pregnancy and birth outcomes in a representative multi-racial/ethnic population with generalizable findings.

## Background

In September 2016, the National Institutes of Health (NIH) awarded 35 centers across the U.S. to participate with their extant cohorts in the pediatric cohort initiative of the Environmental Influences on Child Health Outcomes (ECHO) research program [1]. The ECHO pediatric cohort component of the ECHO program will utilize existing research study cohort populations around the nation aiming to enroll more than 50,000 children to investigate how exposure to a range of environmental factors in early development - from conception through early childhood - influences the health of children and adolescents [1].

Environmental factors that might have affected the health of children and adolescents during the last decades include synthetic chemicals. Owing to the exponential rise in the development and production of synthetic chemicals since the 1940s, the global community has experienced an unprecedented level of chemical exposures. Pregnant women and developing fetuses are no exception. Animal data and emerging, yet limited epidemiological data suggest the particular vulnerability of the pregnant population and developing fetuses to perturbation by chemicals, as illustrated by adverse pregnancy and birth outcomes and their long-term consequences [2, 3]. As such, the NIH and the Environmental Protection Agency have funded several pregnancy cohorts since 1998 [4], and recently the ECHO pediatric cohort, which comprises 84 extant cohorts. One of the extant cohorts included in ECHO is an ongoing prospective birth cohort named the Pregnancy Environment and Lifestyle Study (PETALS). Funded by the National Institute of Environmental Health Sciences in 2013, PETALS aims to enroll 3,350 pregnant women in northern California to investigate whether exposure to synthetic chemicals, such as phenols, early in pregnancy was associated with increased risk of developing gestational diabetes (GDM) and the related birth outcome, neonatal macrosomia.

The incidence of GDM has increased by more than 35% during the past decades [5], which may have been fueling the epidemic of obesity and type 2 diabetes among both women and their offspring [6]. Mirroring the escalating epidemic of GDM is the widespread use of endocrine disrupting chemicals (EDCs), which have been implicated in obesity and altered glucose homeostasis [7]. One such class of EDCs includes the phenols. However, epidemiological data on phenol exposure, particularly from prospective studies with multiple measurements during pregnancy and measures of potential mechanistic metabolic biomarkers are extremely scarce. Further, given that phenols can cross the maternal blood-placental barrier [8], investigation on their potential impact on fetal growth and birth outcomes is warranted. However, such prospective, intergenerational human data are limited.

As such, we launched the PETALS to address these critical data gaps. This paper describes the design, research activities, and cohort profile from October 2013 to February 2016 in the ongoing PETALS.

## Methods/design

The scope of PETALS is to: 1) establish a longitudinal birth cohort of 3,350 mother-infant pairs with fasting blood and urine collection at the first and second trimesters and extensive questionnaire data, and 2) conduct a nested case–control study of 300 women with GDM and 600 matched controls to determine: a) the association between early pregnancy urinary levels of phenols and the risk of developing GDM; b) whether early pregnancy urinary levels of phenols are associated with adverse changes in glucose metabolism, markers of insulin resistance, and liver function; and c) whether markers of insulin resistance and/or markers of liver function mediate the association between phenols and GDM.

### Study design

The current research activity of PETALS is a longitudinal pregnancy cohort, within which a nested case–control study of phenols and GDM risk is underway. Women are enrolled from the Kaiser Permanente Northern California (KPNC) membership. Fasting blood draw, urine collection, and questionnaires are completed at enrollment and follow-up with targeted windows of 10–13 and 16–19 weeks of gestation, respectively. Women’s electronic health records (EHR) can be abstracted from the year prior to the index pregnancy to delivery, and their children’s birth outcomes and health information can be obtained from the EHR.

### Setting and study population

The PETALS is conducted at KPNC, which is an integrated health care delivery system serving 3.6 million members, representing approximately 30% of the population across 14 counties of Greater Bay Area, as well as the California Central Valley from Sacramento to Fresno. Across 16 delivery hospitals, KPNC provides prenatal care to approximately 38,000 pregnancies annually. The population is racially/ethnically and socio-economically diverse and highly representative of the entire population living in the served geographic area [9, 10]. KPNC maintains complete real-time databases on all hospitalizations, outpatient visits, laboratory tests, and medications through the EHR, which make identification of women at the beginning of their pregnancy very efficient. For PETALS, we focus recruitment at medical centers with racial and ethnic diversity to obtain a large number of GDM cases. At these medical centers, we have built a strong relationship with obstetricians and perinatologists who allow us to conduct research and study clinic visits in the exam rooms where study participants receive their medical care. Study blood draw and urine collection are performed at the clinical laboratory within the medical centers.

### Eligibility and enrollment

The PETALS scans for positive pregnancy tests and first prenatal visits on a weekly basis. Upon identification, pregnant women of all races/ethnicities, aged 18–45 years, with a gestational age less than 11 weeks, and who are able to provide informed consent in English are sent a recruitment letter and contacted by phone calls to determine eligibility. Exclusion criteria include multiple gestations or any of the following conditions diagnosed prior to conception: diabetes, cancer, hepatitis C, or liver cirrhosis. If eligible, the research assistant calls to schedule the baseline clinic visit and sends a packet containing a consent form, questionnaires, blood collection instructions, facility map, and a reminder letter by mail or email, upon ascertaining participant’s preferred modality. Women have the option to sign informed consent and complete questionnaires online. Women are further excluded if termination of pregnancy, diagnosis of diabetes, or use of diabetes medication occurred before the baseline clinic examination. The study has been approved by the human subjects committee of the Kaiser Foundation Research Institute.

### Data collection and clinic visits

Upon participants’ consent, the research assistant places an order in HealthConnect (KPNC’s integrative electronic health record management system) for a urine collection and a fasting blood draw at the baseline clinic visit (10–13 weeks of gestation). Women are instructed to fast for 8 h prior to the blood draw and the duration since last time eating/drinking (except water) is recorded at each visit. The research assistant meets the participant in the morning between 8:00 and 11:00 am at her medical center and first reviews the informed consent whether or not already signed online and asks participants if they need any further clarifications. Women who had signed the consent online are also asked to sign on paper during the visit, for our record. The baseline study clinic visit includes urine specimen collection, fasting blood draw, anthropometric measurements, and several self-administered questionnaires if not completed prior to the visit (either online or on paper).

Similarly, the second study clinic visit is targeted at 16–19 weeks of gestation (at least 6 weeks apart from the baseline study clinic visit), at which time urine and fasting blood samples and a survey assessing environmental exposures to phenols are obtained. Urine and blood specimens are transferred by couriers in the standard climate controlled containers along with the biospecimen samples collected for routine clinical care to the KPNC Clinical Regional Laboratory and then to the Kaiser Permanente Research Bank (KPRB) Biorepository.

#### Blood collection and storage

**Fig. 1:**
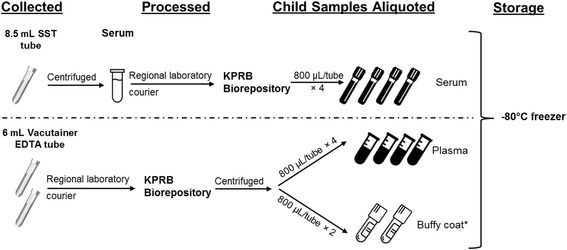
Prenatal blood processing protocol for PETALS. ^*^For future DNA extraction

At both clinic visits, fasting blood samples are collected in one 8.5 mL serum separator tube (SST) and two 6.0 mL ethylenediaminetetraacetic acid (EDTA) tubes. The SST is centrifuged within 30 min of blood collection at the medical center’s clinical laboratory. Once at the KPRB Biorepository, serum from the 8.5 mL SST tube is aliquoted into four, 0.8 mL cryovials; the 6 mL Vacutainer EDTA tube is centrifuged and plasma is aliquoted into four, 0.8 mL cryovials, while buffy coat is aspirated and placed in two, 0.8 mL cryovials. All cryovials are stored at −80 °C and are linked to subjects with barcode labels (Fig. 1).

#### Urine collection and storage

**Fig. 2:**
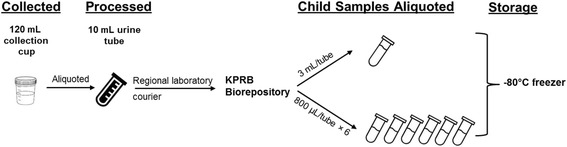
Prenatal urine processing protocol for PETALS

To increase the generalizability of phenol exposure, urine specimens are collected during a “typical day”, as defined by participants, as was done previously [11]. Urine collection follows the Centers for Disease Control and Prevention protocol [12]. At each study visit, women are provided with a 120 mL capacity collection cup free of bisphenol-A (BPA) and a sealable plastic biohazard bag. The time of urine collection and the time they last emptied their bladder are recorded. A 10 mL urine sample is aliquoted from each urine collection cup and transferred via regional laboratory couriers in a tube free of BPA. Once at the KPRB Biorepository, urine samples are aliquoted into one, 3 mL cryovial until phenol assays are performed within four months, and six, 0.8 mL cryovials for future studies. All cryovials are stored at −80 °C and are linked to subjects with barcode labels (Fig. 2).

#### Quality control procedures

PETALS developed a rigorous protocol to reduce external chemical contamination in the collection process. Women are instructed not to use a wipe prior to urine collection since wipes may contain the phenol triclosan (TCS); women and study staff are asked not to use sunscreen or lotions on the day of the study visit since they may contain bisphenol-3 (BP-3). On a semi-annual basis, all the collection materials (polypropylene cups, tubes, pipettes, etc.) at each study medical center and all materials at the biorepository are tested by the California State Biomonitoring Laboratory to ensure they are BPA free. Every three months we also run procedure field blanks, which entails running a solution of blank water (OmniSolv LC-MS Water) through our collection process and then the California State Biomonitoring Laboratory tests the blank water to ensure it is BPA, BP-3 and TCS free. So far, collection and processing materials and procedure blanks have shown no detectable contamination by BPA, BP-3, or TCS via this protocol. This protocol will be in place until the end of the study.

#### BPA exposure questionnaire

While direct measurement of urinary BPA levels will define BPA exposure, we also assess the common sources of exposures (i.e., the consumption of food and beverages related to BPA) via the Environmental Assessment survey administered at study clinic visit 2 but not visit 1, in order to decrease the possibility that participants might make behavioral changes due to increased attention to potential sources of exposure after completing the survey.

#### Maternal anthropometry

At clinic visit 1, weight and height are measured with a standard, calibrated scale (Tanita WB-110 A, Tokyo, Japan) and stadiometer according to standard anthropometric protocols, respectively [13]. While the participant is standing erect with her abdomen relaxed, waist circumference is measured by positioning a tape one inch above the umbilicus at the end of the participant’s normal expiration; hip measurement is obtained at the maximum extension of the buttocks. Each measurement is taken in duplicate. The mean value of each anthropometric measurement is calculated if the two initial measurements agree within 1 cm for length and circumference or within 1 lb (0.45 kg) for weight. Otherwise, an additional measurement is taken and the third recording will be used. Pre-pregnancy weight is obtained by abstracting the weight measured closest to the last menstrual period within 12 gestational weeks prior from the EHR and by self-reported values if EHR data are missing (less than 2%). Pre-pregnancy body mass index is calculated as pre-pregnancy weight (kg), divided by squared height (m^2^) measured at the baseline clinic visit. The last weight measured within 3 weeks before delivery is abstracted from the EHR and used to calculate gestational weight gain by subtracting pre-pregnancy weight.

#### Food and nutrient intake

To assess women’s habitual diet in early pregnancy, we use the Block food frequency questionnaire (FFQ) [14], focusing on the time period between conception and baseline clinic visit. The FFQ is supplemented with an abbreviated set of questions to assess prenatal vitamins and supplements. The Block FFQ has been validated and used in numerous study populations including pregnant women [15].

#### Physical activity

A validated pregnancy physical activity questionnaire is administered to assess physical activity during the previous two months prior to baseline clinic visit [16], supplemented with questions about moderate and vigorous physical activity within one year before pregnancy.

#### Other lifestyle factors

Data on sleep duration and quality during and before pregnancy are obtained via the baseline questionnaire. In addition to questions on maternal and household tobacco use and maternal alcohol consumption before and during pregnancy, data on maternal stress via the Patient Health Questionnaire-(8) [17] and depression during pregnancy via the 10-item Perceived Stress Scale [18] are collected at baseline study visit.

#### Other covariates

Information on women’s race/ethnicity, age, education, income, parity, and family history of diabetes is obtained by baseline questionnaire; information on women’s medical history can be extracted from the EHR. In addition, women are asked to complete a questionnaire to provide information on father of the baby including race/ethnicity, weight, and height.

#### Infant outcomes

Infant birthweight and length are collected from the EHR. Gestational age at birth is based on the woman’s expected delivery date extracted from the EHR and back calculated last menstrual period date. Large for gestational age is defined as a birthweight greater than the gestational age-, sex-, and racial/ethnical-specific 90^th^ percentile, and small for gestational age as less than the 10^th^ percentile for the KPNC population’s gestational age and racial/ethnical specific birthweight distribution as utilized previously [19]. Macrosomia is defined as a birthweight ≥4000 g.

### Additional resources

#### KPNC clinical databases and EHRs

KPNC has maintained clinical records in electronic format for several decades. In 2005, KP HealthConnect, a fully integrated EHR system designed by the Epic Corporation, was implemented in everyday care delivery with real-time data. All aspects of members’ clinical care, encounters and communications, and administrative and demographic data are integrated and available in HealthConnect. In addition, a single, consolidated reporting data repository, the Virtual Data Warehouse, is sourced from the KP HealthConnect Operational EHR data repository, updated nightly, and used for reporting, research, and analytics. The Virtual Data Warehouse is a set of standardized research datasets maintained by KPNC, with standardized file definitions, that includes virtually all of the clinical data in the EHR.

#### Possible linkage with environmental and area-level databases and census data

Environmental and area-level data can be linked to participants, largely by leveraging available public use datasets and geographic information system methods. First, “clinical environmental” factors, such as exposure to medical radiation and pharmaceuticals, are captured in the EHR. Second, the residential address of each member has been geocoded and provides coordinates to link to geospatial data including US census data from 1970 to current releases of the American Community Survey data (e.g., income/poverty, education, employment, housing, and occupation). We have obtained data from the California Environmental Protection Agency (CalEnviroScreen), Air Resources Board (e.g., air quality and the Toxic Releases Inventory), and Department of Pesticide Regulation (California Pesticide Use Report data), which can be linked to the study cohort.

### Prospective nested case–control study of phenols and GDM risk

#### GDM case definition and identification

At KPNC, 96% of pregnancies are screened for GDM with the 50-g, 1-h glucose challenge test (GCT) at 24–28 weeks of gestation [20], thus after the second PETALS research clinic visit at 16–19 weeks. If the screening test is abnormal, a diagnostic 100-g, 3-h oral glucose tolerance test (OGTT) is performed in the morning after a 12-h fast. Plasma glucose measurements are performed using the hexokinase method at the KPNC regional laboratory, which participates in the College of American Pathologists' accreditation and monitoring program.

GDM cases are identified by searching the glucose results from the 50-g, 1-h GCT and the 100-g, 3-h OGTT during pregnancy. If a woman underwent more than one screening or diagnostic test, we use the tests performed closest to her delivery. In order to be selected as a GDM case, a woman must have performed the GCT and meets any of the following criteria currently used at KPNC: 1) a fasting glucose ≥92 mg/dL performed alone or during the 100-g, 3-h OGTT, as recommended by the International Association of Diabetes and Pregnancy Study Groups diagnostic criteria [21]; or 2) two or more plasma glucose values after the 100-gram oral glucose load meeting or exceeding the Carpenter and Coustan thresholds: 1-h 180 mg/dL, 2-h 155 mg/dL, and 3-h 140 mg/dL [22]. We anticipate to identify 300 GDM cases when we will reach the sample size of 3,350 women enrolled in the longitudinal birth cohort.

#### Control selection and matching criteria

Among women without an indication of GDM as defined above, two controls for each case are selected and individually matched to each case using information from the EHR and study questionnaire, leading to a total of 600 controls. Specifically, we first restrict our sample of possible controls to women who have delivered and were screened for GDM via the 50-g, 1-h GCT to ensure complete ascertainment of their pregnancy glycemia and we then exclude those who had one abnormal glucose value after the 100-gram oral glucose load (1-h ≥180 mg/dL, 2-h ≥155 mg/dL, and 3-h ≥140 mg/dL) to ensure a greater distinction between cases and controls. Then as soon as a case is selected, within each participating medical center, potential controls individually matched to the case on self-reported race/ethnicity, age (±5 years), calendar time of enrollment (±3 months), and gestational weeks at clinic visit 1 (±3 weeks) are identified. The two best matched controls with the smallest weighted sum over matching variables of absolute case–control difference were selected for each case.

#### Urine phenols and creatinine assay

Levels of urinary phenols in GDM cases and controls are measured by the California State Biomonitoring Laboratory. BPA is measured using high-pressure liquid chromatography tandem mass spectrometry as described by Gavin et al. [23]. Urine samples are processed by enzymatic deconjugation with β-glucuronidase/sulfatase, followed by solid phase extraction on a C18 cartridge, evaporation of the solvent, and reconstitution with mobile phase eluant. Analytes are separated by reversed-phase high performance liquid chromatography-isotope dilution tandem mass spectrometry. Laboratory quality assurance includes systematic internal and external proficiency testing. Method blanks, as well as absolute and relative recovery, are monitored during sample preparation and analysis to ensure they are within acceptable ranges. Quality control materials are run with each batch of study samples to verify method precision and accuracy. The California State Biomonitoring Laboratory also analyzes duplicate samples from 5% of subjects for additional precision assessment. We also submit 2% of “blind” duplicates to the State laboratory to provide an independent quality control in addition to the quality control run by the State laboratory. Urinary creatinine concentrations are also measured in order to appropriately adjust phenols levels for urinary creatinine levels, according to a method previously described [24].

#### Potential mediators of the phenols-GDM association

Emerging experimental data suggests that BPA specifically during pregnancy may adversely affect glucose and insulin metabolism [25], potentially via alterations in adiponectin secretion [26] and liver enzymes [27]. Therefore, we assess markers of glucose and insulin metabolism and liver enzymes as potential mediating factors of the association between BPA exposure and risk of GDM.

Serum samples of GDM cases and matched controls from the baseline visit and the second trimester visit are sent to the Lipid and Apolipoprotein laboratory at the University of Washington, Seattle, WA. Glucose is measured with an oxidation reaction using a glucose analyzer (YSI 2300 STAT Plus; YSI, Yellow Springs, OH). Insulin is measured using the Millipore radioimmunoassay St Charles MO). Adiponectin is measured by a commercially available radioimmunoassay (Millipore) using 125I-labeled murine adiponectin and a multispecies anti-adiponectin antibody; measurements are performed in duplicate and results are reported as the mean. Liver enzymes alanine aminotransferases (ALT) and gamma-glutamyl transferase (GGT) are measured on a Polychem analyser (PolyMedCo Inc., Cortlandt Manor, NY). All assays are performed without knowledge of GDM status.

#### Sample size and statistical power

Given the observed incidence of GDM (9%) in 2010 at the participating KPNC facilities, we anticipate to identify 300 GDM cases out of 3,350 pregnancies during the study period. We computed the minimum detectable odds ratios based on the likelihood ratio test of the association between a multi-level exposure variable and GDM in the context of a logistic regression analysis and a case–control study of 300 GDM cases and 600 controls [28, 29]. We assumed an analysis of quartiles of BPA in relation to GDM, assuming a graded, linear trend in (log) odds ratios across categories and a test for trend. We hypothesize that increased BPA levels will be associated with an increased risk of GDM. The sample size will provide 80% power to detect an OR of 1.71 for the association between the highest BPA quartile and the risk of GDM for a two-sided test at level 0.05.

In summary, we collect research quality clinical data, questionnaires, and biospecimens for the measurements shown in Table 1 from all women. Notably, as specified in the table, measurements of BPA, BP-3, TCS, insulin, glucose, adiponectin, GGT, and ALT are going to be performed among 300 GDM cases and 600 controls to conduct a prospective case–control study nested within the entire PETALS cohort, as an efficient approach for relatively rare outcomes and expensive exposure assessments.

**Table 1:** Overview of data collection for expected 3350 women in the Pregnancy and Environment Lifestyle Study (PETALS)

	Pregnancy			Delivery	Data source
	10–13 weeks	16–19 weeks	24–28 weeks		
Maternal variables					
Demographics					
Age	X				Survey
Race/ethnicity	X				Survey
Household SES	X				Survey
Medical history	X	X	X	X	Survey/EHR
Reproductive history	X				Survey/EHR
Behavioral/lifestyle					
Maternal diet by FFQ	X				Survey [14]
Physical activity	X				Survey [16]
Sleep	X				Survey
Smoking and alcohol	X				Survey
Stress and depression	X				Survey [17, 18]/EHR
Anthropometry					
Pre-pregnancy weight	X				EHR
Weight	X				CV
Height	X				CV
Waist and hip circumferences	X				CV
Gestational weight gain	X	X	X	X	EHR
Self-reported products use		X			Survey
Biospecimen collection					
Fasting blood	X	X			
Urine	X	X			
GDM ascertainment			X		EHR
50-g, 1-h GCT			X		EHR
100-g, 3-hour OGTT			X		EHR
Infant variables					
Estimated fetal size				X	EHR
Birth size (weight, length)				X	EHR
Gestational age at delivery				X	EHR
Infant sex				X	EHR
Measurements for the phenols-GDM case control study^a^					
Urinary BPA/BP-3/TCS levels					
Glucose, insulin, HOMA-IR	X^a^	X^a^			Serum
Total adiponectin	X^a^	X^a^			Serum
Liver enzymes (ALT and GGT)	X^a^	X^a^			Serum

*ALT* aminotransferases, *BPA* bisphenol-A, B*P-3* bisphenol-3, *CV* clinic visit, *EHR* electronic health record, *FFQ* food frequency questionnaire, *GCT* glucose challenge test, *GGT* gamma-glutamyl transferase, *HOMA-IR* homeostatic model assessment-insulin resistance, *OGTT* oral glucose tolerance test, *SES* socioeconomic status, *TCS* triclosan

^a^Measured among 300 expected GDM cases and 600 controls

## Interim results

Between October 2013 and February 2016, 2,416 women who initiated prenatal care with a gestation age <11 weeks at a KPNC medical center participating in the PETALS were deemed eligible via telephone interview and enrolled in the study. As of February 2016, 1,823 women had completed the baseline clinic visit, leading to a participation rate of 75%. Further, 1,653 completed a follow-up clinic visit (with 97 being scheduled for a future date and 32 having become ineligible due to fetal loss or serious complications after the baseline visit), leading to a retention rate of 98%.

As of February 29, 2016, a total of 1415 participants had delivered and their characteristics are shown in Table 2. Overall, the PETALS cohort exhibits racial/ethnic diversity with 24.1% non-Hispanic White, 41.1% Hispanic, 21.1% Asian, 10.1% African American, and 3.6% of other races/ethnicities. The majority (84.9%) had an education level above high school. Approximately 60% were multiparous and 60.9% were overweight or obese before pregnancy. A total of 149 (10.5%) women had GDM according to the criteria described above. On average, infants were delivered at 38.8 (SD 2.0) weeks of gestation with a mean birthweight of 3362.9 (SD 538.3) g.

**Table 2:** Characteristics of participants who had delivered as of February 29, 2016 in the PETALS (*n* = 1,415)

Age, years, n (%)	
18–24	251 (17.7)
25–29	377 (26.6)
30–34	515 (36.4)
35–39	234 (16.5)
40–44	38 (2.7)
Race/Ethnicity, n (%)	
Non-Hispanic White	341 (24.1)
Hispanic	582 (41.1)
Asian	298 (21.1)
African American	143 (10.1)
Other	51 (3.6)
Education, n (%)	
High school or less	210 (14.8)
Some College	568 (40.1)
College graduate	386 (27.3)
Post graduate	247 (17.5)
Unknown	4 (0.3)
Household income, $, n (%)	
< 50,000	487 (34.4)
50,000-99,999	467 (33.0)
100,000-149,999	241 (17.0)
≥ 150,000	191 (13.5)
Unknown	29 (2.0)
Parity, n (%)	
0	587 (41.5)
1	522 (36.9)
≥ 2	287 (20.3)
Unknown	19 (1.3)
Pre-pregnancy BMI, kg/m^2^, n (%)	
< 18.5	37 (2.6)
18.5–24.9	515 (36.4)
25.0–29.9	436 (30.8)
≥ 30.0	426 (30.1)
Unknown	1 (0.1)
Gestational age at study visit, weeks, mean (SD)	
Clinic visit 1	13.6 (2.3)
Clinic visit 2	19.8 (2.5)
Gestational diabetes, n (%)	149 (10.5)
Gestational age at delivery, weeks, mean (SD)	38.8 (2.0)
Infant birthweight, g, mean (SD)	3362.9 (538.3)

## Discussion

We report on the rationale, objectives, and data collection of the PETALS prospective pregnancy cohort and present the cohort profile among participants enrolled from October 2013 and who delivered as of February 2016. We will prospectively examine the association between prenatal exposure to BPA, BP-3 and TCS and subsequent risk of GDM, as well as the associations between urinary phenols and metabolic markers of insulin resistance and liver enzymes in a nested case–control study of expected 300 GDM cases and 600 matched controls, to elucidate the mechanisms by which phenols affect glucose homeostasis during pregnancy.

As highlighted recently, several organizations including the American College of Obstetricians and Gynecologists [30], the American Society for Reproductive Medicine [30], and International Federation of Gynecology and Obstetrics [31] have joined leading scientists and called for timely action to prioritize research on toxic environmental chemicals in relation to reproductive health outcomes. Given the ubiquitous nature of these compounds, even a modest increase in the risk of GDM and offspring obesity due to in-utero exposure to these chemicals would have significant public health implications. In this regard, findings from the PETALS present a unique opportunity to address timely and urgent public health concerns regarding toxic environmental chemicals in relation to reproductive and fetal health outcomes during the critical window of pregnancy and fetal development.

The PETALS has several notable strengths. First, it will include a large and racially/ethnically diverse cohort of 3,350 pregnant women who will provide urine and blood specimens, as well as data on behavioral/lifestyle factors. The diverse demographic characteristics may enhance the generalizability of our findings. With a wealth of data on major socioeconomic, medical, and lifestyle confounders, we may minimize the impact of residual confounding while assessing the associations of interest. Second, the prospective, nested case–control design will minimize potential for selection and recall biases, addressing the study hypotheses in a cost-effective manner. Third, the use of two measurements of BPA, BP-3, and TCS during the first and second trimesters will allow us to reduce intra-individual variability and better assess phenol exposures. Moreover, data on metabolic markers of insulin resistance and liver enzymes offer an opportunity to investigate the potential mechanisms underlying a potential association between BPA and GDM risk. Besides maternal outcomes, we will be able to explore the association between prenatal phenol exposure and neonatal outcomes based on data extracted from the EHR, which may provide insights into the intergenerational impact of intrauterine exposure to phenols on offspring health outcomes.

Finally, PETALS’s data could also be linked and extended to a myriad of additional clinical and observational data. Specifically, the study will provide an opportunity to investigate the associations between intrauterine environmental exposures and other pregnancy complications such as hypertensive disorders during pregnancy (i.e., gestational hypertension, preeclampsia, and eclampsia) and adverse birth outcomes such as preterm birth and neonatal complications. This is of great relevance given that GDM may enhance the risk of pregnancy-induced hypertensive complications [32] and neonatal morbidity [33] . In addition, stored biospecimens at the state-of-the-art KPRB Biorepository may provide opportunities to examine additional environmental chemicals including other EDCs (perfluoroalkyl substances, polybrominated diphenyl ethers, and contemporary organophosphate flame retardants) in relation to infant growth and neurodevelopment as we proposed for the ECHO study. We are requesting additional funding to explore the association between intrauterine phenol exposure and fetal and infant growth and fat accrual during the first year of life as well as to explore how maternal metabolic substrates, such as lipids, free fatty acids, and thyroid hormones may affect fetal growth and infant growth, a unique model to study the early origins of obesity. Furthermore, the KPNC setting provides a unique ability to follow up women and their infants, which allows for opportunities to investigate the longitudinal and intergenerational impact of intrauterine exposure to environmental chemicals on maternal and child health consequences in later life, respectively.

In summary, the ongoing PETALS collects, processes, and stores urine and blood specimens from pregnant women during the first and second trimesters, which will be linked to extensive data from questionnaires, clinical measurements, and the EHR. Further, as one of the birth cohorts included in the ECHO pediatric initiative, by leveraging and combining extant multiple types of data (socio-demographic, chemical, lifestyle and behavioral, clinical, and diagnostic) in the PETALS with future follow-up, this multi-component research infrastructure could provide unique opportunities to address critical questions on the role of the intrauterine environment in the developmental origins of disease.

## Abbreviations

ALT: Alanine aminotransferases
BP-3: Bisphenol-3
BPA: Bisphenol-A
ECHO: Environmental influences on child health outcomes
EDTA: Ethylenediaminetetraacetic acid
EHR: Electronic health records
FFQ: Frequency questionnaire
GCT: Glucose challenge test
GDM: Gestational diabetes
GGT: Gamma-glutamyl transferase
KPNC: Kaiser Permanente Northern California
KPRB: Kaiser Permanente Research Bank
NIH: National Institutes of Health
OGTT: Oral glucose tolerance test
PETALS: Pregnancy environment and lifestyle study
SST: Serum separator tube
TCS: Triclosan

## References

[1] NIH awards more than $150 million for research on environmental influences on child health. ECHO program to investigate exposures from conception through early childhood. [https://www.nih.gov/news-events/news-releases/nih-awards-more-150-million-research-environmental-influences-child-health]. Accessed 8 Apr 2017.

[2] Meeker JD (2012). Exposure to environmental endocrine disruptors and child development. Arch Pediatr Adolesc Med.

[3] Gore AC, Chappell VA, Fenton SE, Flaws JA, Nadal A, Prins GS, Toppari J, Zoeller RT (2015). EDC-2: The Endocrine Society's Second Scientific Statement on Endocrine-Disrupting Chemicals. Endocr Rev.

[4] A Decade of Children’s Environmental Health Research: Highlights from EPA’s Science to Achieve Results Program. [https://www.epa.gov/sites/production/files/2015-09/documents/decade-of-childrens-health-full-report.pdf]. Accessed 8 Apr 2017.

[5] Ferrara A (2007). Increasing prevalence of gestational diabetes mellitus: a public health perspective. Diabetes Care.

[6] Zhu Y, Zhang C (2016). Prevalence of Gestational Diabetes and Risk of Progression to Type 2 Diabetes: a Global Perspective. Curr Diabetes Rep.

[7] Thayer KA, Heindel JJ, Bucher JR, Gallo MA (2012). Role of environmental chemicals in diabetes and obesity: a National Toxicology Program workshop review. Environ Health Perspect.

[8] Balakrishnan B, Henare K, Thorstensen EB, Ponnampalam AP, Mitchell MD (2010). Transfer of bisphenol A across the human placenta. Am J Obstet Gynecol.

[9] Krieger N (1992). Overcoming the absence of socioeconomic data in medical records: validation and application of a census-based methodology. Am J Public Health.

[10] Gordon N, Lin T (2016). The Kaiser Permanente Northern California Adult Member Health Survey. Perm J.

[11] Li DK, Odouli R, Wi S, Janevic T, Golditch I, Bracken TD, Senior R, Rankin R, Iriye R (2002). A population-based prospective cohort study of personal exposure to magnetic fields during pregnancy and the risk of miscarriage. Epidemiology.

[12] Blood and urine collection. [http://www.cdc.gov/nchs/data/nhanes/nhanes_09_10/labcomp_f.pdf]. Accessed 8 Apr 2017.

[13] Lohman T, Roche A, Martorell R (1988). Anthropometric standardization reference manual.

[14] Block G, Hartman AM, Dresser CM, Carroll MD, Gannon J, Gardner L (1986). A data-based approach to diet questionnaire design and testing. Am J Epidemiol.

[15] Harley K, Eskenazi B, Block G (2005). The association of time in the US and diet during pregnancy in low-income women of Mexican descent. Paediatr Perinat Epidemiol.

[16] Chasan-Taber L, Schmidt MD, Roberts DE, Hosmer D, Markenson G, Freedson PS (2004). Development and validation of a Pregnancy Physical Activity Questionnaire. Med Sci Sports Exerc.

[17] Spitzer RL, Kroenke K, Williams JB: Validation and utility of a self-report version of PRIME-MD: the PHQ primary care study. Primary Care Evaluation of Mental Disorders. Patient Health Questionnaire. JAMA : the journal of the American Medical Association. 1999;282(18):1737-44. 10.1001/jama.282.18.1737 10568646

[18] Cohen S, Kamarck T, Mermelstein R: A global measure of perceived stress. J Health Soc Behav. 1983;24(4):385-96. 6668417

[19] Ehrlich SF, Crites YM, Hedderson MM, Darbinian JA, Ferrara A (2011). The risk of large for gestational age across increasing categories of pregnancy glycemia. Am J Obstet Gynecol.

[20] Ferrara A, Kahn HS, Quesenberry CP, Riley C, Hedderson MM (2004). An increase in the incidence of gestational diabetes mellitus: Northern California, 1991–2000. Obstet Gynecol.

[21] Metzger BE, Gabbe SG, Persson B, Buchanan TA, Catalano PA, Damm P, Dyer AR, Leiva A, Hod M, International Association of Diabetes and Pregnancy Study Groups Consensus Panel (2010). International association of diabetes and pregnancy study groups recommendations on the diagnosis and classification of hyperglycemia in pregnancy. Diabetes Care.

[22] American College of Obstetricians and Gynecologists Committee on Practice Bulletins--Obstetrics: ACOG Practice Bulletin, American College of Obstetricians and Gynecologists Committee on Practice Bulletins--Obstetrics (2001). ACOG Practice Bulletin. Clinical management guidelines for obstetrician-gynecologists. Number 30, September 2001 (replaces Technical Bulletin Number 200, December 1994). Gestational diabetes. Obstet Gynecol.

[23] Gavin QW, Ramage RT, Waldman JM, She J (2014). Development of HPLC-MS/MS method for the simultaneous determination of environmental phenols in human urine. Int J Environ Anal Chem.

[24] Barr DB, Wilder LC, Caudill SP, Gonzalez AJ, Needham LL, Pirkle JL (2005). Urinary creatinine concentrations in the U.S. population: implications for urinary biologic monitoring measurements. Environ Health Perspect.

[25] Alonso-Magdalena P, Garcia-Arevalo M, Quesada I, Nadal A (2015). Bisphenol-A treatment during pregnancy in mice: a new window of susceptibility for the development of diabetes in mothers later in life. Endocrinology.

[26] Hugo ER, Brandebourg TD, Woo JG, Loftus J, Alexander JW, Ben-Jonathan N (2008). Bisphenol A at environmentally relevant doses inhibits adiponectin release from human adipose tissue explants and adipocytes. Environ Health Perspect.

[27] Bindhumol V, Chitra KC, Mathur PP (2003). Bisphenol A induces reactive oxygen species generation in the liver of male rats. Toxicology.

[28] Shieh G (2000). On power and sample size calculations for likelihood ratio tests in generalized linear models. Biometrics.

[29] Statistics and Epidemiology Research Corporation. EGRET SIZ Reference Manual. (Manual revision 10). Seattle: Statistics and Epidemiology Research Corporation; 1992.

[30] American College of Obstetricians and Gynecologists (2013). Exposure to toxic environmental agents. Committee Opinion No. 575. Obstet Gynecol.

[31] Di Renzo GC, Conry JA, Blake J, DeFrancesco MS, DeNicola N, Martin JN, McCue KA, Richmond D, Shah A, Sutton P (2015). International Federation of Gynecology and Obstetrics opinion on reproductive health impacts of exposure to toxic environmental chemicals. Int J Gynaecol Obstet.

[32] Bryson CL, Ioannou GN, Rulyak SJ, Critchlow C (2003). Association between gestational diabetes and pregnancy-induced hypertension. Am J Epidemiol.

[33] Persson B, Hanson U (1998). Neonatal morbidities in gestational diabetes mellitus. Diabetes Care.

